# Stability and physical compatibility of parenteral nalbuphine hydrochloride during continuous infusion in pediatrics

**DOI:** 10.1371/journal.pone.0330869

**Published:** 2025-09-04

**Authors:** Romain Paoli-Lombardo, Vincent Arcani, Delphine Dazan, Caroline Castera-Ducros, Marie-Anne Esteve, Marjorie Roudot, Chloé Wanert, Caroline Ovaert, Christophe Curti, Patrice Vanelle, Pascal Rathelot

**Affiliations:** 1 Service Central de la Qualité et de l’Information Pharmaceutiques (SCQIP), Pharmacy Department, AP-HM, Hôpital de la Conception, Marseille, France; 2 CNRS, Institut de Chimie Radicalaire ICR, UMR, Equipe de Pharmaco-Chimie Radicalaire, Aix Marseille Univ, Marseille, France; 3 Aix Marseille Univ, AP-HM, Hôpital de la Timone, Pharmacy Department, Marseille, France; 4 Service médico-chirurgical de cardiologie pédiatrique et congénitale, AP-HM, Hôpital de la Timone, Marseille, France; University of Messina, ITALY

## Abstract

In pediatric practice, nalbuphine hydrochloride can be administered by continuous infusion through a multiport manifold or Y-site connection over 24 h in a 60 mL polypropylene syringe unprotected from light at concentrations ranging from 52.1 µg.mL^-1^ to 333.3 µg.mL^-1^ in normal saline (NS). To limit the need for multiple injections, nalbuphine hydrochloride may be co-administered with other drugs. Its stability and compatibility were already studied at concentration ranges equal or higher than 1.0 mg.mL^-1^. However, when nalbuphine hydrochloride needs to be diluted for a pediatric administration its stability in NS over 24 h of light exposure at ambient temperature and its compatibility with other drugs have never been studied before. A novel chromatographic method using high-performance liquid chromatography (HPLC) was validated by the International Council for Harmonisation (ICH) Q2 (R1) guidelines. The stability of nalbuphine hydrochloride and the appearance of degradation products under five experimental conditions (light, heat, oxidation, basicity, and acidity) were monitored by HPLC-UV for 24 h at ambient temperature at three concentrations administered in pediatric departments (52.1 µg.mL^-1^, 166.7 µg.mL^-1^ and 333.3 µg.mL^-1^). The physical compatibility of nalbuphine hydrochloride with 1:1 (v/v) mixtures of selected drugs used in pediatrics was evaluated by visual inspection and with a 10 µm and 25 µm sub-visible particle counter. Our nalbuphine hydrochloride quantification method has been validated and was stability-indicating. The stability of nalbuphine hydrochloride and the forced degradation assay (light, heat, oxidation, basicity, and acidity) studied for the three concentrations of nalbuphine hydrochloride diluted in 48 mL of NS and stored in a 60 mL polypropylene syringe unprotected from light were compliant for at least 24 h at ambient temperature. Nalbuphine hydrochloride in NS was found to be compatible with several drugs, but was incompatible with furosemide and amphotericin B. Nalbuphine hydrochloride can be administered in pediatric practice using a syringe pump for 24 h. However, drug-drug incompatibilities need to be considered when it is administered through a multiport manifold or Y-site connection.

## Introduction

Nalbuphine hydrochloride is a synthetic phenanthrene opioid, agonist of κ receptors, and antagonist of μ receptors, with analgesic and sedative properties. It does not cause respiratory depression, and it is therefore frequently prescribed in pediatric cardiology departments for pain management [1]. In our hospital, low doses of nalbuphine hydrochloride ranging from 2.5 mg to 16 mg in 48 mL of NS (concentrations ranging from 52.1 µg.mL^-1^ to 333.3 µg.mL^-1^) are administered by continuous infusion through a multiport manifold or Y-site connection over 24 h using a 60 mL polypropylene syringe unprotected from light.

These low-dose syringes are prepared from Nalbuphine Renaudin®, a high-dose injectable solution of 20 mg nalbuphine hydrochloride in 2 mL NS (concentration of 10 mg.mL^-1^), which is not suitable for direct pediatric administration. Before use, Nalbuphine Renaudin® can be stored for 3 years at ambient temperature and must be protected from light. The literature shows that a preparation of 1 mg.mL^-1^ of nalbuphine hydrochloride in NS was stable for one year under refrigerated conditions [2]. However, the stability of low doses of nalbuphine hydrochloride in NS over 24 h of light exposure at ambient temperature has never been studied.

A novel chromatographic method using HPLC-UV was developed to quantify and determine the stability of nalbuphine hydrochloride. The stability of nalbuphine hydrochloride and the appearance of degradation products under five experimental conditions (light, heat, oxidation, basicity, and acidity) were monitored at three concentrations administered in pediatric departments (52.1 µg.mL^-1^, 166.7 µg.mL^-1^ and 333.3 µg.mL^-1^) in NS for 24 h at ambient temperature, and were evaluated using several appropriate parameters (variation of nalbuphine concentration and formation of degradation products by HPLC, sterility, pH, the visual appearance of the solution and number of sub-visible particles). Finally, the physical compatibility of nalbuphine hydrochloride with several parenteral drugs frequently used in the pediatric population was evaluated by visual inspection and with a 10 µm and 25 µm sub-visible particle counter.

## Materials and methods

Nalbuphine hydrochloride syringes were prepared as in pediatric practice. Normal saline (NS) used for dilution came from 0.9% w/w commercial sodium chloride sterile solution (Fresenius®). The highest concentration (16 mg/48mL = 333.3 µg.mL^-1^) was prepared with 1.60 mL of a commercial 20 mg/2 mL Nalbuphine Renaudin® and NS qs (quantum satis) 48 mL in a 60 mL polypropylene syringe (Ref 002022970F, Penta 60 LL, Pentaferte). The syringe was connected to a polyethylene tubing (71100.20 diam 1−2 mm V-green extension, Vygon®) and placed in a syringe pump (Pilote C IEC, Fresenius®). The flow rate was set at 2 mL.h^-1^ during the stability study, and sampling was performed at the extremity of the tubing. Syringes were unprotected from light and stored at ambient temperature. The other two concentrations (8 mg/48 mL = 166.7 µg.mL^-1^ and 2.5 mg/48 mL = 52.1 µg.mL^-1^) were prepared according to the same protocol, using respectively 0.80 mL and 0.25 mL of a commercial 20 mg/2 mL Nalbuphine Renaudin® diluted with NS qs 48 mL.

The nalbuphine hydrochloride dosing method was performed with a Dionex UltiMate 3000 HPLC-UV (Thermo Fisher Scientific, Waltham, MA, USA) on a Stability® 100 Basic C18 column (250 x 4.6 mm; 5 µm particle size) heated at 50 °C. The mobile phase consisted of 70% phase A, which was a 5 mM sodium acetate trihydrate (Thermo Scientific) buffer prepared in ultrapure water obtained from a water purification system (Direct-Q® 3 UV, Merck Millipore) adjusted to pH 5.5 with diluted acetic acid (VWR), and 30% phase B composed of acetonitrile (Fischer Chemical). The flow was set at 0.8 mL.min^-1^ in isocratic mode, injection volume at 20 µL, and wavelength at 284 nm.

The method was validated by the ICH Q2 (R1) guidelines. The linearity of the process was determined using six calibration curves. Each calibration curve was established with seven different concentrations (25 µg.mL^-1^, 50 µg.mL^-1^, 100 µg.mL^-1^, 200 µg.mL^-1^, 300 µg.mL^-1^, 400 µg.mL^-1^ and 500 µg.mL^-1^) prepared from commercial 20 mg/2 mL Nalbuphine Renaudin® in the mobile phase. The repeatability and the intermediate precision were evaluated with nalbuphine hydrochloride pharmaceutical standard (CERN-051, LGC Standards, 1.0 mg.mL^-1^ in methanol, certified reference material), at three concentrations (40 µg.mL^-1^, 200 µg.mL^-1^ and 400 µg.mL^-1^), using the within-day relative standard deviation (RSD) and the between-day RSD, respectively. The RSD values had to be < 5% for each concentration to be validated. The accuracy was assessed by measuring the bias between mean values and true values at three concentrations (40 µg.mL^-1^, 200 µg.mL^-1^and 400 µg.mL^-1^). For each concentration, the bias values had to be < 5% to have an acceptable accuracy.

The method was validated to be stable, as indicated by a forced degradation study performed under five experimental conditions (light, heat, oxidation, basicity and acidity), according to the ICH Q2 (R2) guidelines. For light conditions, nalbuphine hydrochloride was placed under a sunlamp, and for thermal stress, the solution was placed in an oven at 80 °C. For oxidative conditions, 30% hydrogen peroxide was added. For basic conditions, 1 M sodium hydroxide (VWR Chemicals) was used, neutralized with 0.75 M hydrochloric acid (VWR Chemicals) just before analysis. For acidic conditions, 2 M hydrochloric acid (VWR Chemicals) was used, neutralized with 2 M sodium carbonate (EMSURE® ISO, Sigma-Aldrich) just before analysis. The final concentration of the five samples was 100 µg.mL^-1^. The stability of each concentration was tested in triplicate (three syringes) for 24 h. Sampling points were performed at T0, 2 h, 4 h, 6 h, 8 h, 12 h and 24 h after dilution. Nalbuphine hydrochloride content and degradation product’s appearance or increase were evaluated at each sampling point in triplicate.

For stability studies performed before marketing authorization of commercial specialties, Active Pharmaceutical Ingredient (API) content has to remain between 95% and 105% of its initial value, as specified by the ICH [3]. However, this limit does not apply to “real-life” stability studies performed on reconstituted or diluted commercial specialty (such as nalbuphine) or on compounded drugs. In the literature, several authors [4] and scientific societies [5] recommend to consider a drug to be stable when the API content remains between 90% and 110% of its initial value. Without any authoritative source, this value is open to debate, and some authors recommend the 95–105% limit for cytotoxic drugs [6]. However, in the literature, the wide majority of published real-life stability studies deals with a stability limit of 90–110% for API content.

A sterility test was performed at T0 and 24 h after dilution with a membrane filtration protocol. Briefly, 40 mL of nalbuphine solution were added in 300 mL of sterile diluent + tween 80 (Becton Dickinson, Le Pont-de-Claix, France). Sterile diluent was filtered with a sterility test system (Sterisart®, containers reference: 16477-GBD, Sartorius). Then, one container was filled with 100 mL of fluid thioglycollate medium (Becton Dickinson) and the second was filled with 100 mL of TSB medium (Biomérieux, Marcy-l’Étoile, France). Samples were put in two incubators for 14 days at 25 °C and 35 °C. When it was realized for the first time, applicability of this sterility test was demonstrated under the protocol described in the European Pharmacopoeia with six reference strains (Biomérieux Bioball®): *Staphylococcus aureus* NCTC10788, *Bacillus subtilis* NCTC10400, *Pseudomonas aeruginosa* NCTC12924, *Candida albicans* NCPF3179, *Aspergillus braziliensis* NCPF2275 and *Clostridium sporogenes* NCTC532. pH was measured once at each sampling point with the FiveEasy pH meter F20 (Mettler Toledo). Visual examination of the solution was performed at each sampling point by a single qualified technician. Samples were visually explored for particles contamination or color change under an artificial light exposure in front of a white and black panel. Sub-visible particles were checked in triplicate with the HIAC 9703 + liquid particle counter (Beckman Coulter) at each sampling point, according to assay 1.B of the European Pharmacopoeia. For each sample container of 48 mL, the number of sub-visible particles had to remain below 6000 particles larger than 10 µm and below 600 particles larger than 25 µm for 24 h to have an acceptable stability.

Finally, nalbuphine compatibility was studied with 1:1 (v/v) mixtures of selected drugs at concentrations used in pediatrics: acyclovir (5 mg.mL^-1^, NS), amikacin (2.5 mg.mL^-1^, NS), amoxicillin (20 mg.mL^-1^, NS), amphotericin B (0.5 mg.mL^-1^, Dextrose 5% in Water D5W), cefotaxime (20 mg.mL^-1^, NS), clonazepam (0.04 mg.mL^-1^, NS), diazepam (0.02 mg.mL^-1^ and 1 mg.mL^-1^, NS), dobutamine (1 mg.mL^-1^, NS), dopamine (1.6 mg.mL^-1^, NS), furosemide (1 mg.mL^-1^, NS or D5W), midazolam (0.04 mg.mL^-1^, NS), sodium bicarbonate (14 mg.mL^-1^) and vancomycin (5 mg.mL^-1^, D5W). Nalbuphine hydrochloride compatibility with NS after adjustment of pH to 3, 5, 7, 9 or 11 was also studied. Visual examination as well as 10 µm and 25 µm sub-visible particle counting (HIAC 9703 + Beckman Coulter) were performed at T0, 5 min, and 30 min after 1:1 (v/v) mixture with respectively 2.5 mg/48 mL, 8 mg/48 mL, 16 mg/48mL nalbuphine hydrochloride in NS and pure NS as negative control. The compatibility of nalbuphine hydrochloride with 1:1 (v/v) mixtures of selected drugs used in pediatrics and with pH values was evaluated according to assay 1.B of the European Pharmacopoeia. For each sample container of 96 mL, the number of sub-visible particles had to remain below 6000 particles larger than 10 µm and below 600 particles larger than 25 µm for 24 h to have an acceptable stability.

## Results

**Fig 1:**
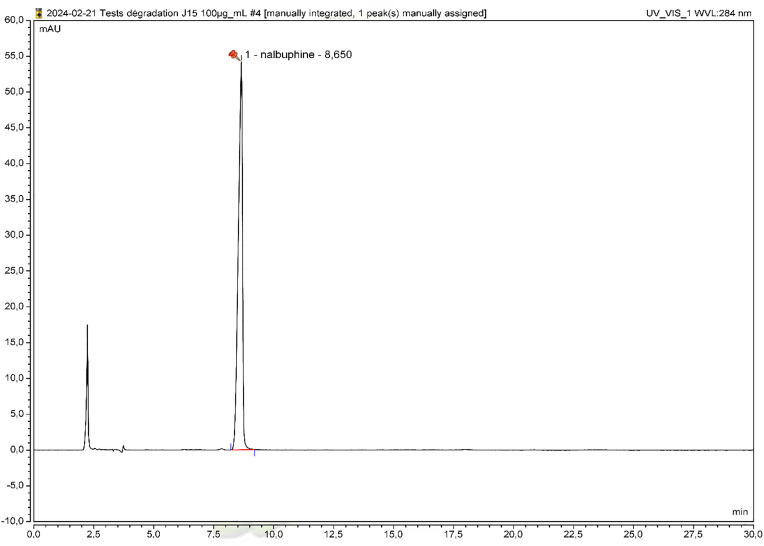
Nalbuphine hydrochloride chromatogram.

Under our analytical conditions, nalbuphine hydrochloride was identified as a single symmetrical peak with a retention time (RT) of 8.65 min (Fig 1).

The HPLC-UV method for nalbuphine hydrochloride quantification was validated. The linearity of the process was demonstrated between 25 µg.mL^-1^ and 500 µg.mL^-1^ (R^2^ > 0.99 and deviation < 10% for each concentration). Repeatability, intermediate precision and accuracy were confirmed with within-day RSD and between-day RSD values <5% and bias values <5% (Table 1).

**Table 1:** Repeatability, intermediate precision, and accuracy.

Samples	Repeatability n = 15 (% RSD within-day)	Intermediate precision n = 18 (% RSD between-day)	Accuracy n = 18 (Bias in %)
Nalbuphine hydrochloride 40 µg.mL^-1^	1.56%	3.71%	2.50%
Nalbuphine hydrochloride 200 µg.mL^-1^	2.00%	1.76%	1.08%
Nalbuphine hydrochloride 400 µg.mL^-1^	0.86%	0.79%	1.11%

The quantification method was also found to indicate stability. The results of the forced degradation study as a synthetic table and the forced degradation study’s chromatograms are respectively shown in the S1 Table and S1–S6 Figs. It can be noticed that nalbuphine hydrochloride has a retention time between 8.3 and 8.6 min under all experiments, except under alkaline conditions, where retention time was shifted at 10.5 min, probably because nalbuphine hydrochloride ionization state was altered.

After method validation and forced degradation experiments, stability studies were performed. The nalbuphine hydrochloride solutions remained stable over 24 h for each parameter studied. Monitoring of pH variation during a stability study is a classical, simple measurement which can reflect a gaz diffusion though the drug’s container and/or a drug degradation yielding to pH change (for example, with nalbuphine, though its amino- or alcohol- groups) [6,7]. Here, the pH in the nalbuphine syringes did not vary by more than 10% over 24 h for the three concentrations tested (Table 2).

**Table 2:** pH of nalbuphine hydrochloride solutions over time during stability studies.

	0	2 h	4 h	6 h	8 h	12 h	24 h
52.1 µg.mL^-1^ (2.5 mg/48 mL)	4.43	4.11	4.04	4.29	4.04	4.03	4.07
166.7 µg.mL^-1^ (8 mg/48 mL)	3.91	3.88	3.79	3.87	3.81	3.84	3.84
333.3 µg.mL^-1^ (16 mg/48 mL)	3.80	3.86	3.80	3.74	3.77	3.82	3.76

**Fig 2:**
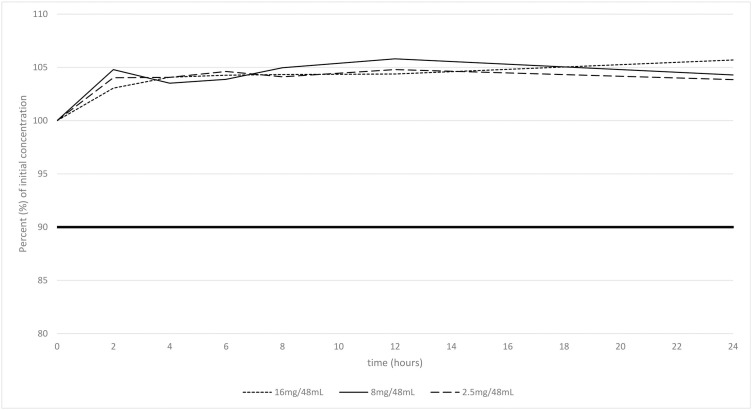
Evolution of nalbuphine content in polypropylene syringes over 24 h.

For each sample, the number of sub-visible particles remained below 6000 particles larger than 10 µm and 600 particles larger than 25 µm for 24 h (assay 1.B of European Pharmacopoeia). Nalbuphine hydrochloride content also remained stable (above 90% but also 95% of the initial nalbuphine hydrochloride content) for 24 h at each concentration tested, without the appearance of new peaks that could correspond to degradation products (Fig 2). Plots of the 95% confidence interval for the evolution of nalbuphine at each concentration tested in polypropylene syringes over 24 h using the normal distribution are shown in S7-S9 Figs.

All syringes were initially sterile and remained sterile during our study.

Table 3 presents the results of the nalbuphine compatibility study, which included both visual inspection and sub-visible particle counting immediately (T0), 5 min (T5) and 30 min (T30) after mixing. Nalbuphine alone did not evidenced either visible or sub-visible particles (Table 3, line 1) at the three tested concentrations. We found nalbuphine hydrochloride compatible with all the drugs tested, except furosemide and amphotericin B. Furosemide/ nalbuphine mixtures yielded to a visible precipitate irrespective of the nalbuphine concentration and the furosemide diluent.

**Table 3:** Compatibility study of nalbuphine hydrochloride with 1:1 (v/v) mixtures of selected drugs used in pediatrics.

	Normal Saline	Nalbuphine 52.1 µg.mL^-1^	Nalbuphine 166.7 µg.mL^-1^	Nalbuphine 333.3 µg.mL^-1^
Control	T0 = C T5 = C T30 = C	T0 = C T5 = C T30 = C	T0 = C T5 = C T30 = C	T0 = C T5 = C T30 = C
Acyclovir (5 mg.mL^-1^, NS)	T0 = C T5 = C T30 = C	T0 = C T5 = C T30 = C	T0 = C T5 = C T30 = C	T0 = C T5 = C T30 = C
Amikacin (2.5 mg.mL^-1^, NS)	T0 = C T5 = C T30 = C	T0 = C T5 = C T30 = C	T0 = C T5 = C T30 = C	T0 = C T5 = C T30 = C
Amoxicillin (20 mg.mL^-1^, NS)	T0 = C T5 = C T30 = C	T0 = C T5 = C T30 = C	T0 = C T5 = C T30 = C	T0 = C T5 = C T30 = C
Amphotericin B (0.5 mg.mL^-1^, D5W)	T0 = **NCI** T5 = **NCI** T30 = **NCI**	T0 = **NCI** T5 = **NCI** T30 = **NCI**	T0 = **NCI** T5 = **NCI** T30 = **NCI**	T0 = **NCI** T5 = **NCI** T30 = **NCI**
Cefotaxime (20 mg.mL^-1^, NS)	T0 = C T5 = C T30 = C	T0 = C T5 = C T30 = C	T0 = C T5 = C T30 = C	T0 = C T5 = C T30 = C
Clonazepam (0.04 mg.mL^-1^, NS)	T0 = C T5 = C T30 = C	T0 = C T5 = C T30 = C	T0 = C T5 = C T30 = C	T0 = C T5 = C T30 = C
Diazepam (0.02 mg.mL^-1^, NS)	T0 = C T5 = C T30 = C	T0 = C T5 = C T30 = C	T0 = C T5 = C T30 = C	T0 = C T5 = C T30 = C
Diazepam (1 mg.mL^-1^, NS)	T0 = C T5 = C T30 = C	T0 = C T5 = C T30 = C	T0 = C T5 = C T30 = C	T0 = C T5 = C T30 = C
Dobutamine (1 mg.mL^-1^, NS)	T0 = C T5 = C T30 = C	T0 = C T5 = C T30 = C	T0 = C T5 = C T30 = C	T0 = C T5 = C T30 = C
Dopamine (1.6 mg.mL^-1^, NS)	T0 = C T5 = C T30 = C	T0 = C T5 = C T30 = C	T0 = C T5 = C T30 = C	T0 = C T5 = C T30 = C
Furosemide (1 mg.mL^-1^, NS)	T0 = C T5 = C T30 = C	T0 = **NCV/I** T5 = **NCV/I** T30 = **NCV/I**	T0 = **NCV/I** T5 = **NCV/I** T30 = **NCV/I**	T0 = **NCV/I** T5 = **NCV/I** T30 = **NCV/I**
Furosemide (1 mg.mL^-1^, D5W)	T0 = C T5 = C T30 = C	T0 = **NCV/I** T5 = **NCV/I** T30 = **NCV/I**	T0 = **NCV/I** T5 = **NCV/I** T30 = **NCV/I**	T0 = **NCV/I** T5 = **NCV/I** T30 = **NCV/I**
Midazolam (0.04 mg.mL^-1^, NS)	T0 = C T5 = C T30 = C	T0 = C T5 = C T30 = C	T0 = C T5 = C T30 = C	T0 = C T5 = C T30 = C
Sodium bicarbonate (14 mg.mL^-1^)	T0 = C T5 = C T30 = C	T0 = C T5 = C T30 = C	T0 = C T5 = C T30 = C	T0 = C T5 = C T30 = C
Vancomycin (5 mg.mL^-1^, D5W)	T0 = C T5 = C T30 = C	T0 = C T5 = C T30 = C	T0 = C T5 = C T30 = C	T0 = C T5 = C T30 = C

C = compatible; NCV/I = not compatible, both visually and for sub-visible particles; NCI = not compatible for sub-visible particles only.

Under our conditions, nalbuphine hydrochloride demonstrated physical compatibility across a broad pH range (pH 3–11), with no visible changes or increase in sub-visible particles observed. Detailed results of this experiment are provided in the S2 Table.

## Discussion

Our nalbuphine hydrochloride dosing method has been validated and was stability-indicating. In the literature, we found that only one forced degradation study had been performed before our work [8]. The influence of heat, basic, acidic, and oxidizing conditions was evaluated, and the authors only reported 100% degradation with the appearance of a single peak at Relative Retention Time (RRT) of 0.40 min when nalbuphine was exposed to drastic oxidizing conditions (50% H_2_O_2_ under reflux for 6 h). No degradation of nalbuphine was observed in experiments performed under heat, acid, and basic conditions. In another study [9], the quantification method was reported to be stability-indicating, but the authors did not perform a forced degradation study. However, they analyzed a mixture of nalbuphine and its two main degradation products, the dimer 2,2’-bisnalbuphine (RRT approximatively equal to 1.4–1.6 min) and 10-ketonalbuphine (RRT approximatively equal to 1.6–1.8 min). In our study, the forced degradation study pointed out the formation of three major degradation products with RRTs comprised between 1.6 and 2.3 min when nalbuphine was exposed to heat at 80 °C for 15 days (Table 2). It can be hypothesized that one of these products could be 2,2’-bisnalbuphine and/or 10-ketonalbuphine previously described with similar RRTs [8].

All parameters studied for the three concentrations of nalbuphine hydrochloride (52.1 µg.mL^-1^, 166.7 µg.mL^-1^, and 333.3 µg.mL^-1^) diluted in NS and stored under the same conditions as they are administered in pediatric departments, i.e., in a 60 mL polypropylene syringe unprotected from light, complied at least 24 h at ambient temperature. Thus, nalbuphine hydrochloride can be administered in pediatrics by continuous infusion through a multiport manifold or Y-site connection over 24 h in a 60 mL polypropylene syringe unprotected from light at low concentrations.

We have also identified several drug-drug incompatibilities that need to be considered when nalbuphine hydrochloride is administered through a multiport manifold or Y-site connection. Since nalbuphine hydrochloride was found to precipitate easily under basic conditions during the forced degradation study, we decided to investigate its compatibility with alkaline drugs used in pediatrics, such as, acyclovir, amoxicillin, cefotaxime, furosemide and sodium bicarbonate. Among these drugs, acyclovir (5 mg.mL^-1^ in NS) has been described in the literature as compatible with nalbuphine hydrochloride (10 mg.mL^-1^ in NS) [10], which was confirmed by our results. Conversely, nalbuphine hydrochloride (10 mg.mL^-1^) and sodium bicarbonate (14 mg.mL^-1^) have been described in the literature as incompatible [11]. However, under our conditions, nalbuphine hydrochloride (52.1–333.3 µg.mL^-1^) was found to be compatible with sodium bicarbonate (14 mg.mL^-1^), likely due to the lower concentrations of nalbuphine hydrochloride used. To our knowledge, the other binary mixtures have never been studied. Nalbuphine hydrochloride was visually incompatible and had a higher invisible particle count than required with furosemide (1 mg.mL^-1^, NS or D5W) at each concentration tested (52.1 µg.mL^-1^, 166.7 µg.mL^-1^ and 333.3 µg.mL^-1^). Furosemide was tested when diluted with both NS and D5W because these two solvents can be used to dilute furosemide in our hospital. Under our conditions, amoxicillin and cefotaxime were found to be compatible with nalbuphine hydrochloride.

Considering the poor solubility of nalbuphine hydrochloride under basic conditions, we evaluated its physical compatibility with NS for the three pediatric concentrations (52.1 µg.mL^-1^, 166.7 µg.mL^-1^, and 333.3 µg.mL^-1^) after adjusting the pH to 3, 5, 7, 9 or 11. No visual incompatibility or increase in sub-visible particle counting was observed, indicating that nalbuphine remains physically stable under our conditions.

Although midazolam hydrochloride is an acidic drug, it is known to be poorly soluble and has often been described as incompatible in drug-drug mixtures [12–17]. Midazolam (2.5 mg.mL^-1^) has previously been described as visually compatible with nalbuphine hydrochloride (5 mg.mL^-1^) [18]. Visual inspection and sub-visible particle counting confirmed this under our conditions. Dobutamine hydrochloride, dopamine hydrochloride and vancomycin hydrochloride, three other acidic drugs, were also tested and found to be compatible with nalbuphine hydrochloride under our conditions.

We also tested the compatibility of nalbuphine hydrochloride with clonazepam and diazepam as drugs containing a cosolvent [19], such as ethyl alcohol, benzyl alcohol, or propylene glycol, which can easily precipitate if not properly diluted. Nalbuphine hydrochloride (2.5−10 mg.mL^-1^) and diazepam (5 mg.mL^-1^) have been described as visually incompatible [20]. However, under our conditions, nalbuphine hydrochloride (52.1–333.3 µg.mL^-1^) and diazepam (0.02 and 1 mg.mL^-1^) were compatible, likely due to the higher dilution of the drugs. To investigate this discrepancy, we also tested the compatibility of the mixture at concentrations previously reported in the literature: nalbuphine hydrochloride (2.5 mg.mL^-1^) with diazepam (5 mg.mL^-1^). In this case, we clearly observed visual incompatibility, which was confirmed by a higher sub-visible particle count than required. These results corroborate earlier reports and highlight that drug-drug physical incompatibilities are multifactorial phenomena strongly influenced by the concentrations of the compounds involved.

Amphotericin B was also tested, as it is a suspension that is incompatible with extreme pH drugs and electrolytes. Amphotericin B cholesteryl sulfate complex (0.83 mg.mL^-1^, D5W) and nalbuphine hydrochloride (10 mg.mL^-1^) have already been described to be incompatible [13]. We confirmed these results with lower concentrations of amphotericin B (0.5 mg.mL^-1^) in D5W and nalbuphine hydrochloride in NS. However, amphotericin B mixed with NS alone was also identified to be incompatible, suggesting that drug-drug mixtures between amphotericin B in D5W and another drug solubilized in NS should be avoided.

Finally, amikacin sulfate, an aminoside commonly used in pediatric practice, was also tested and found to be compatible with nalbuphine hydrochloride.

Our work suffers from several limitations.

First, visual examination of samples for particles contamination was performed only by one technician, which could yield to an individual bias. However, all our technicians are qualified yearly for turbidimetric visual control. Moreover, visual examination was also completed with sub-visible particles evaluation.

Then, we conducted a stability study over 24 h to reflect the “real-life” administration conditions, where nalbuphine’s syringes are made every day. It could have been interesting to extend stability study to 48-72h to improve the overall data.

During our stability study, an increase in nalbuphine hydrochloride between T0 and T2 was noticed, whatever the concentration was. As samples were aliquoted after a polyethylene tubing to simulate real-life conditions, we supposed that an adsorption phenomenon in the tubing decreased initial nalbuphine hydrochloride concentration, but we tried to investigate without success.

Finally, we studied physical compatibility of nalbuphine with several drugs, but it could have been interesting to study also its chemical compatibility, using nalbuphine HPLC-UV method.

## Conclusion

Our nalbuphine hydrochloride quantification method has been validated by the ICH Q2 (R1) guidelines and was stability-indicating, according to the ICH Q2 (R2) guidelines. Nalbuphine hydrochloride diluted with NS in polypropylene syringes at concentrations ranging from 52.1 µg.mL^-1^ to 333.3 µg.mL^-1^ is stable for 24 h at ambient temperature and without protection from light. This allows its administration in pediatric practice by continuous infusion using a syringe pump for 24 h. However, several drug-drug incompatibilities need to be considered when administered through a multiport manifold or Y-site connection. At the concentrations tested (52.1 µg.mL^-1^, 166.7 µg.mL^-1^, and 333.3 µg.mL^-1^), nalbuphine hydrochloride in NS is incompatible with furosemide (1 mg.mL^-1^), an alkaline drug, and with amphotericin B (0.5 mg.mL^-1^), a drug suspension.

## Supporting information

S1 Table Results of nalbuphine hydrochloride forced degradation study. Bold and underlined products were found as main degradation products (peak area/nalbuphine peak area > 1.00%) under experimental conditions. For example, after 3 h of exposure to 15% H2O2, nalbuphine content decreased from 25% and five degradation products appeared, two of them as main degradation products (RRT 0.27 and RRT 0.39).
(DOCX)

S1 Fig Nalbuphine forced degradation (light irradiation). (DOCX)

S2 Fig Nalbuphine forced degradation (oxidation). (DOCX)

S3 Fig Nalbuphine forced degradation (oxidation enlarged image). (DOCX)

S4 Fig Nalbuphine forced degradation (heat). (DOCX)

S5 Fig Nalbuphine forced degradation (NaOH). (DOCX)

S6 Fig Nalbuphine forced degradation (HCl). (DOCX)

S7 Fig 95% confidence interval for the evolution of nalbuphine at 333.3 µg.mL^-1^ in polypropylene syringes over 24 h. (DOCX)

S8 Fig 95% confidence interval for the evolution of nalbuphine at 166.7 µg.mL^-1^ in polypropylene syringes over 24 h. (DOCX)

S9 Fig 95% confidence interval for the evolution of nalbuphine at 52.1 µg.mL^-1^ in polypropylene syringes over 24 h. (DOCX)

S2 Table Compatibility study of nalbuphine hydrochloride diluted with NS after pH adjustment. (DOCX)

## References

[1] Kubica-Cielińska A, Zielińska M. The use of nalbuphine in paediatric anaesthesia. Anaesthesiol Intensive Ther. 2015;47(3):252–6. doi: 10.5603/AIT.2015.0036 26165241

[2] Hammer C, Perello L, Dory A, Leveque D, Ubeaud-Sequier G. Nalbuphine stability at 1 mg/mL concentration. AAPS Congress - Washington. 2011.

[3] ICH Topic Q 1 A (R2). Stability Testing of New Drug Substances and Products. Available from: https://www.ema.europa.eu/en/ich-q1a-r2-stability-testing-new-drug-substances-drug-products-scientific-guideline

[4] McPherson T, Kolling W, Navarre E. Timing of the initial assay for stability studies. Am J Health Syst Pharm. 2024;81(7):e156. doi: 10.1093/ajhp/zxad281 37947169

[5] Methodological guidelines for stability studies of hospital pharmaceutical preparations. Available from: https://www.gerpac.eu/IMG/pdf/guide_stabilite_anglais.pdf

[6] Bardin C, Astier A, Vulto A, Sewell G, Vigneron J, Trittler R, et al. Guidelines for the practical stability studies of anticancer drugs: a European consensus conference. Ann Pharm Fr. 2011;69(4):221–31. doi: 10.1016/j.pharma.2011.07.002 21840442

[7] Ajdarić J, Ibrić S, Pavlović A, Ignjatović L, Ivković B. Prediction of Drug Stability Using Deep Learning Approach: Case Study of Esomeprazole 40 mg Freeze-Dried Powder for Solution. Pharmaceutics. 2021;13(6):829. doi: 10.3390/pharmaceutics13060829 34204912 PMC8230350

[8] Quarry MA, Williams RC, Sebastian DS. Determination of degradation products in nalbuphine hydrochloride injection by high performance liquid chromatography. J Liq Chromatogr Relat Technol. 1998;21(18):2841–52.

[9] Attia KA, Nassar MW, El-Olemy A. Stability-Indicating HPLC Method for Determination of Nalbuphine Hydrochloride. Int J Res Pharmaceut Biomed Sci. 2014;1:15–22.

[10] Canann D, Tyler LS, Barker B, Condie C. Visual compatibility of i.v. medications routinely used in bone marrow transplant recipients. Am J Health Syst Pharm. 2009;66(8):727–9. doi: 10.2146/ajhp070572 19336832

[11] Aujoulat P, Coze C, Braguer D, Raybaud C. Physicochemical compatibility of methotrexate with co-administered drugs during cancer chemotherapy regimens. J Pharm Clin. 1993;12:31–5.

[12] Swart EL, Mooren RA, van Loenen AC. Compatibility of midazolam hydrochloride and lorazepam with selected drugs during simulated Y-site administration. Am J Health Syst Pharm. 1995;52(18):2020–2. doi: 10.1093/ajhp/52.18.2020 8528872

[13] Trissel LA, Gilbert DL, Martinez JF. Incompatibility and compatibility of amphotericin B cholesteryl sulfate complex with selected other drugs during simulated Y-site administration. Hosp Pharm. 1998;33:284–92.

[14] Mantong ML, Marquardt ED. Visual compatibility of midazolam hydrochloride with selected drugs during simulated Y-site injection. Am J Health Syst Pharm. 1995;52(22):2567–8. doi: 10.1093/ajhp/52.22.2567 8590242

[15] Good PD, Schneider JJ, Ravenscroft PJ. The compatibility and stability of midazolam and dexamethasone in infusion solutions. J Pain Symptom Manage. 2004;27(5):471–5. doi: 10.1016/j.jpainsymman.2004.02.002 15120775

[16] Ayari G, D’Huart E, Vigneron J, Demoré B. Y-site compatibility of intravenous medications commonly used in intensive care units: laboratory tests on 75 mixtures involving nine main drugs. Pharm Technol Hosp Pharm. 2022;7:20220002.

[17] Serrurier C, Chenot ED, Vigneron J, May I, Demoré B. Assessment of injectable drug’s administration in two intensive care units and determination of potential physico-chemical incompatibilities. EJHP Science. 2006;12:96–9.

[18] Forman JK, Souney PF. Visual compatibility of midazolam hydrochloride with common preoperative injectable medications. Am J Hosp Pharm. 1987;44(10):2298–9. doi: 10.1093/ajhp/44.10.2298 3687973

[19] Soltanpour S, Bastami Z, Sadeghilar S, Kouhestani M, Pouya F, Jouyban A. Solubility of clonazepam and diazepam in polyethylene glycol 200, propylene glycol, N-methyl pyrrolidone, ethanol, and water at (298.2 to 318.2) K and in binary and ternary mixtures of polyethylene glycol 200, propylene glycol, and water at 298.2 K. J Chem Eng Data. 2013;58:307–14.

[20] Jump WG, Plaza VM, Poremba A. Compatibility of nalbuphine hydrochloride with other preoperative medications. Am J Hosp Pharm. 1982;39(5):841–3. doi: 10.1093/ajhp/39.5.841 7081259

