# Supplementary material for: Stability and physical compatibility of parenteral nalbuphine hydrochloride during continuous infusion in pediatrics
Source: PLoS One. 2025 Sep 4;20(9):e0330869. doi: 10.1371/journal.pone.0330869 (PMC12410720; doi:10.1371/journal.pone.0330869)
Supplement: S1 Table — Bold and underlined products were found as main degradation products (peak area/nalbuphine peak area > 1.00%) under experimental conditions. For example, after 3 h of exposure to 15% H2O2, nalbuphine content decreased from 25% and five degradation products appeared, two of them as main degradation products (RRT 0.27 and RRT 0.39). (DOCX) [file pone.0330869.s001.docx]

| Reaction condition | % degradation | Degradation products RRT^a^ |
| --- | --- | --- |
| Light irradiation, 15 days (D) | 2% | 0.44; 0.53; 1.23; 1.41; 1.89; 2.04 |
| H_2_O_2_ 15%, 3 h | 25% | **0.27**; 0.36; **0.39**; 0.69; 0.89 |
| 80 ^o^C, 15 D | 28% | 0.36; 0.39; 0.63; 0.66; 0.89; 0.94; **1.58**; **2.10**; **2.29** |
| NaOH 1M, 15 D | 10% | **0.53** |
| HCl 2M, 8 D | 0% | - |

^a^ RRT = Relative Retention Time, defined as the ratio of product’s retention time and nalbuphine retention time.
