# Supplementary figures and images for: Stability and physical compatibility of parenteral nalbuphine hydrochloride during continuous infusion in pediatrics
Source: PLoS One. 2025 Sep 4;20(9):e0330869. doi: 10.1371/journal.pone.0330869 (PMC12410720; doi:10.1371/journal.pone.0330869)

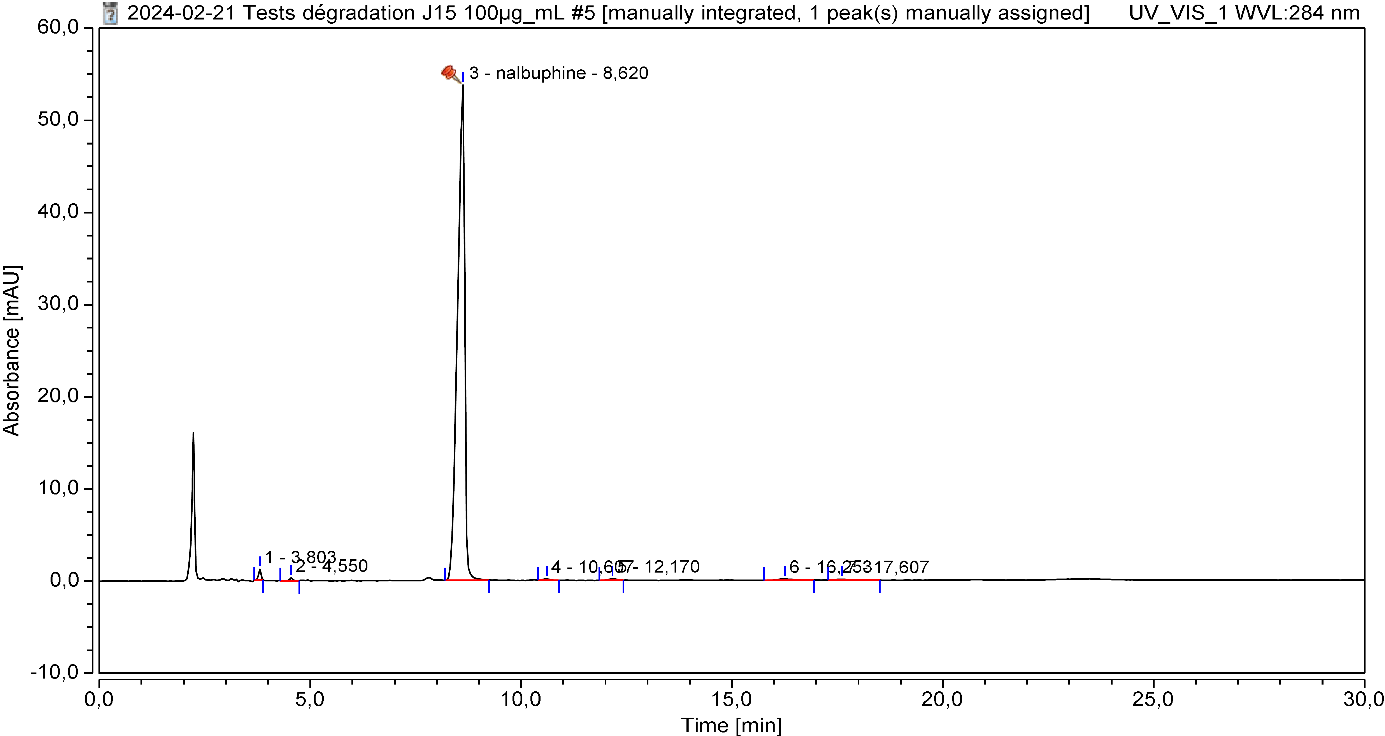

Supplement: S1 Fig — (DOCX) [file pone.0330869.s002.docx]

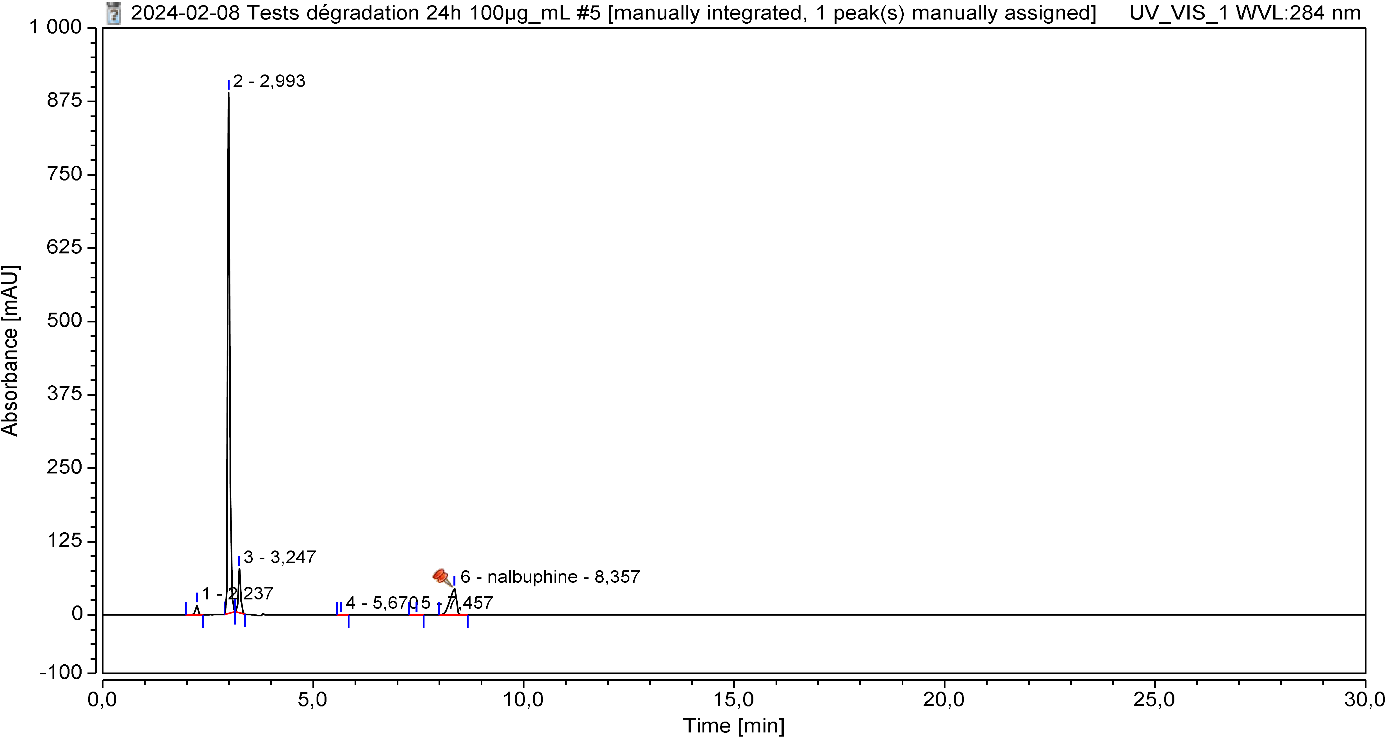

Supplement: S2 Fig — (DOCX) [file pone.0330869.s003.docx]

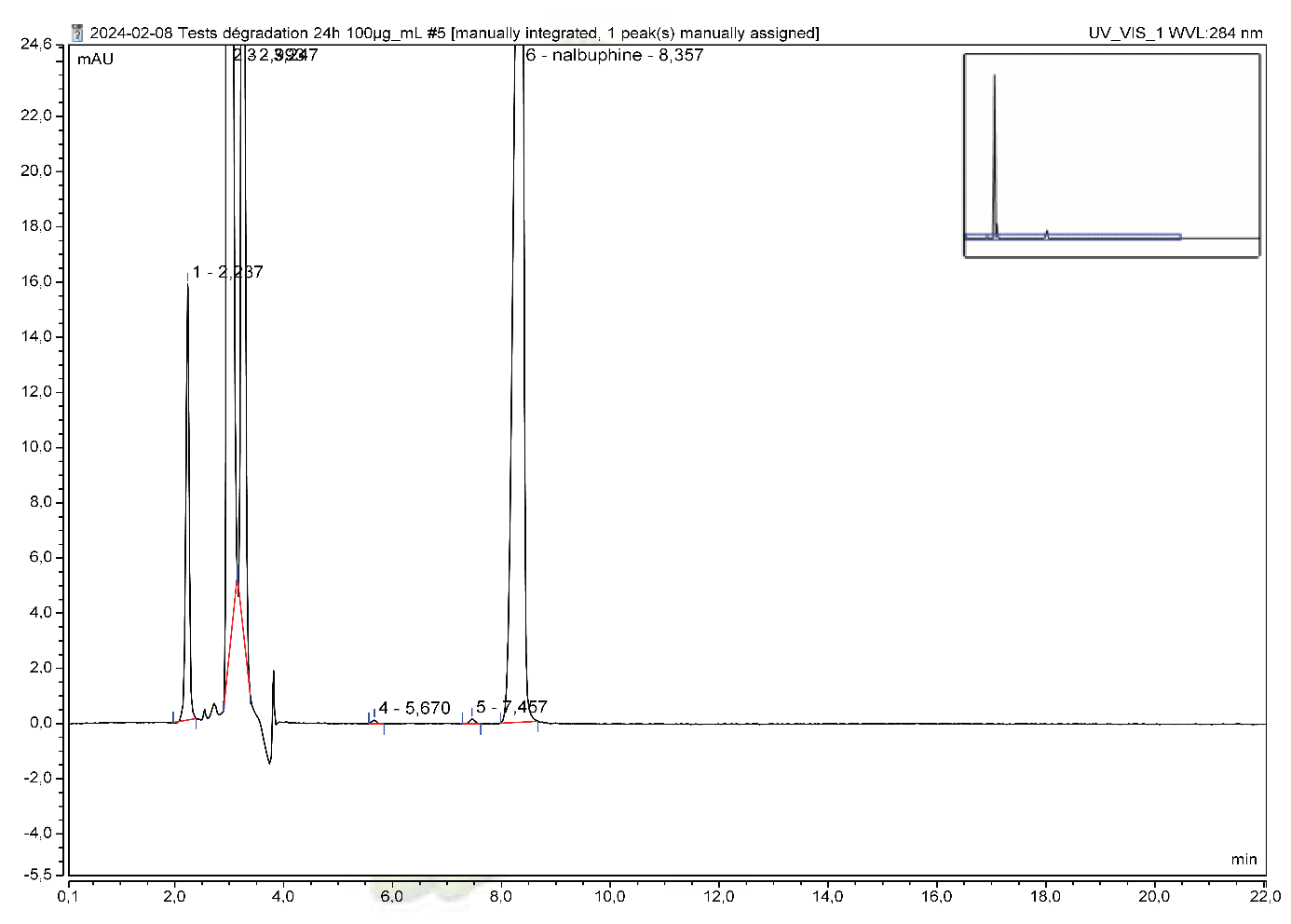

Supplement: S3 Fig — (DOCX) [file pone.0330869.s004.docx]

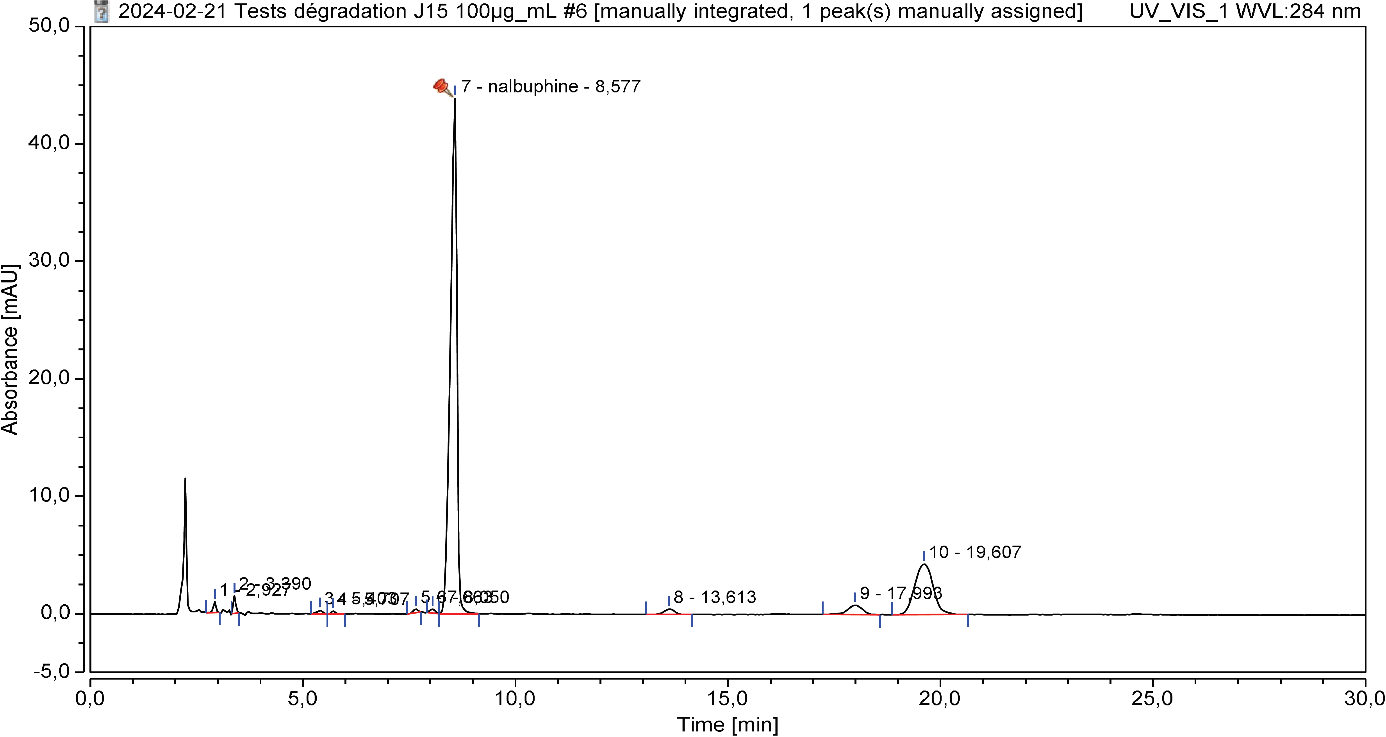

Supplement: S4 Fig — (DOCX) [file pone.0330869.s005.docx]

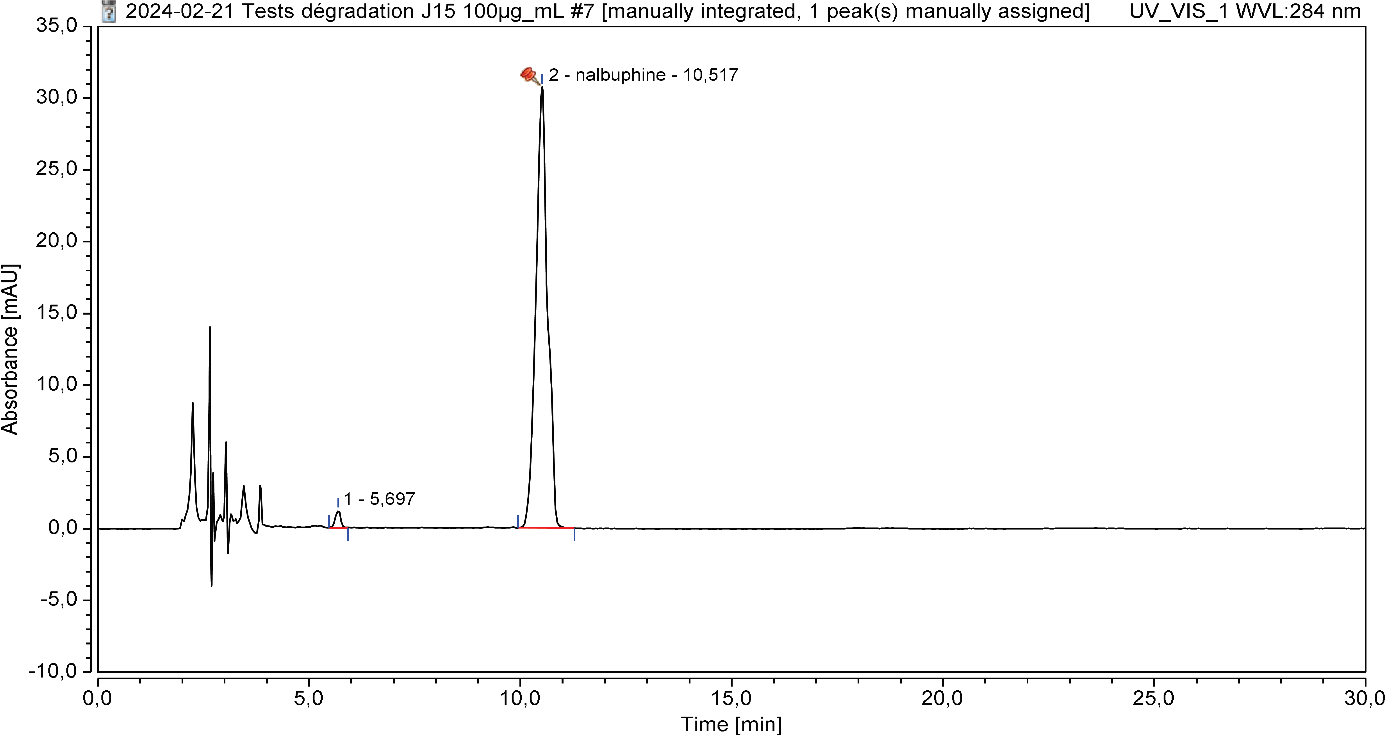

Supplement: S5 Fig — (DOCX) [file pone.0330869.s006.docx]

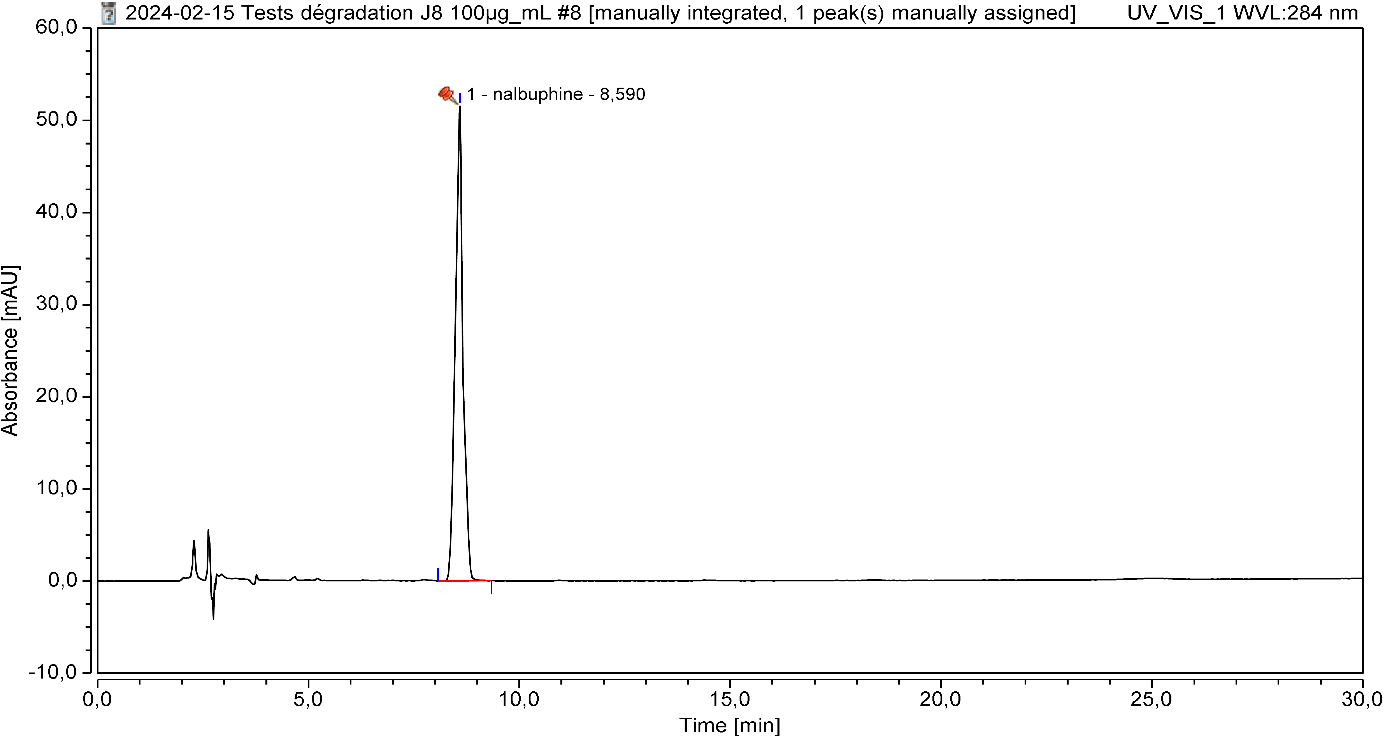

Supplement: S6 Fig — (DOCX) [file pone.0330869.s007.docx]
