# Supplementary material for: Stability and physical compatibility of parenteral nalbuphine hydrochloride during continuous infusion in pediatrics
Source: PLoS One. 2025 Sep 4;20(9):e0330869. doi: 10.1371/journal.pone.0330869 (PMC12410720; doi:10.1371/journal.pone.0330869)
Supplement: S2 Table — (DOCX) [file pone.0330869.s011.docx]

**S2 Table. Compatibility study of nalbuphine hydrochloride diluted with NS after pH adjustment.**

|  | Nalbuphine  52.1 µg.mL^-1^ | Nalbuphine  166.7 µg.mL^-1^ | Nalbuphine  333.3 µg.mL^-1^ |
| --- | --- | --- | --- |
| Normal Saline  (pH 3) | T0 = C  T5 = C  T30 = C | T0 = C  T5 = C  T30 = C | T0 = C  T5 = C  T30 = C |
| Normal Saline  (pH 5) | T0 = C  T5 = C  T30 = C | T0 = C  T5 = C  T30 = C | T0 = C  T5 = C  T30 = C |
| Normal Saline  (pH 7) | T0 = C  T5 = C  T30 = C | T0 = C  T5 = C  T30 = C | T0 = C  T5 = C  T30 = C |
| Normal Saline  (pH 9) | T0 = C  T5 = C  T30 = C | T0 = C  T5 = C  T30 = C | T0 = C  T5 = C  T30 = C |
| Normal Saline  (pH 11) | T0 = C  T5 = C  T30 = C | T0 = C  T5 = C  T30 = C | T0 = C  T5 = C  T30 = C |

C = compatible
