# Supplementary material for: Stability and physical compatibility of parenteral nalbuphine hydrochloride during continuous infusion in pediatrics
Source: PLoS One. 2025 Sep 4;20(9):e0330869. doi: 10.1371/journal.pone.0330869 (PMC12410720; doi:10.1371/journal.pone.0330869)
Supplement: Supplementary file 4 [file pone.0330869.s008.docx]

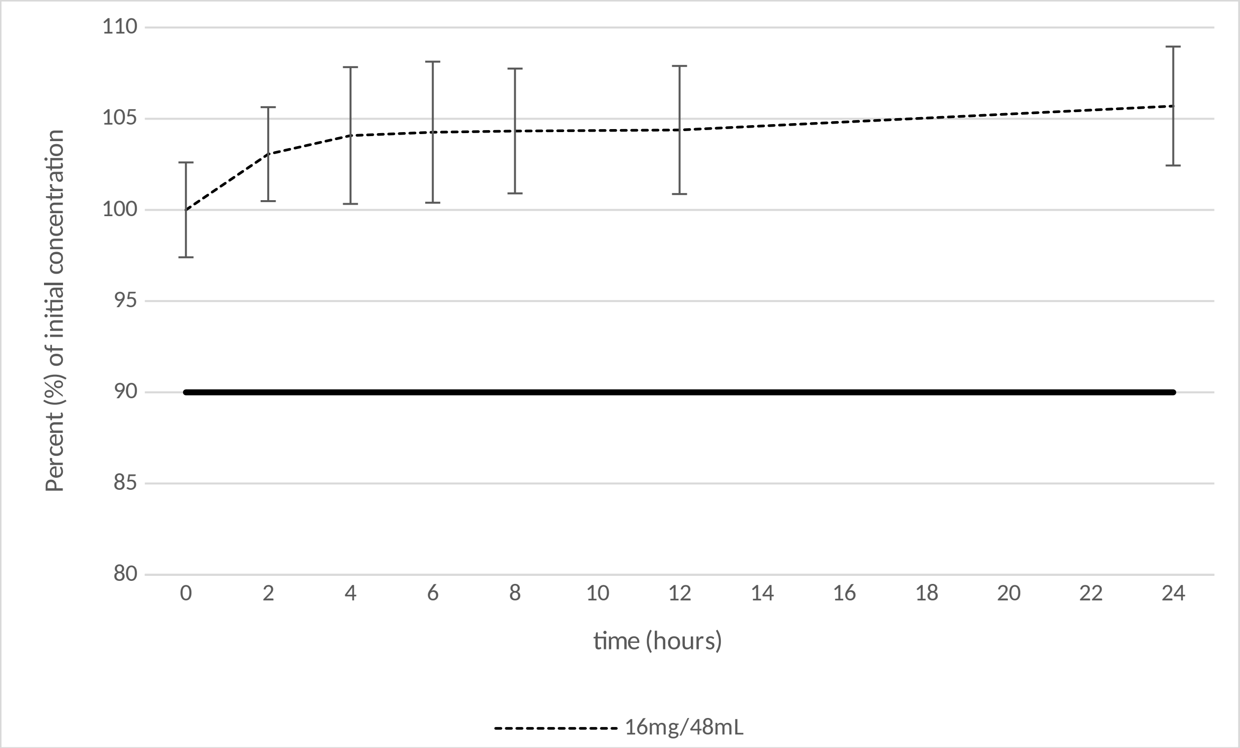
110
105
100
95
90
85
80
Percent (%) of initial concentration
0 2 4 6 8 10 12 14 16 18 20 22 24
time (hours)
16mg/48mL
