# Supplementary material for: Stability and physical compatibility of parenteral nalbuphine hydrochloride during continuous infusion in pediatrics
Source: PLoS One. 2025 Sep 4;20(9):e0330869. doi: 10.1371/journal.pone.0330869 (PMC12410720; doi:10.1371/journal.pone.0330869)
Supplement: Supplementary file 5 [file pone.0330869.s009.docx]

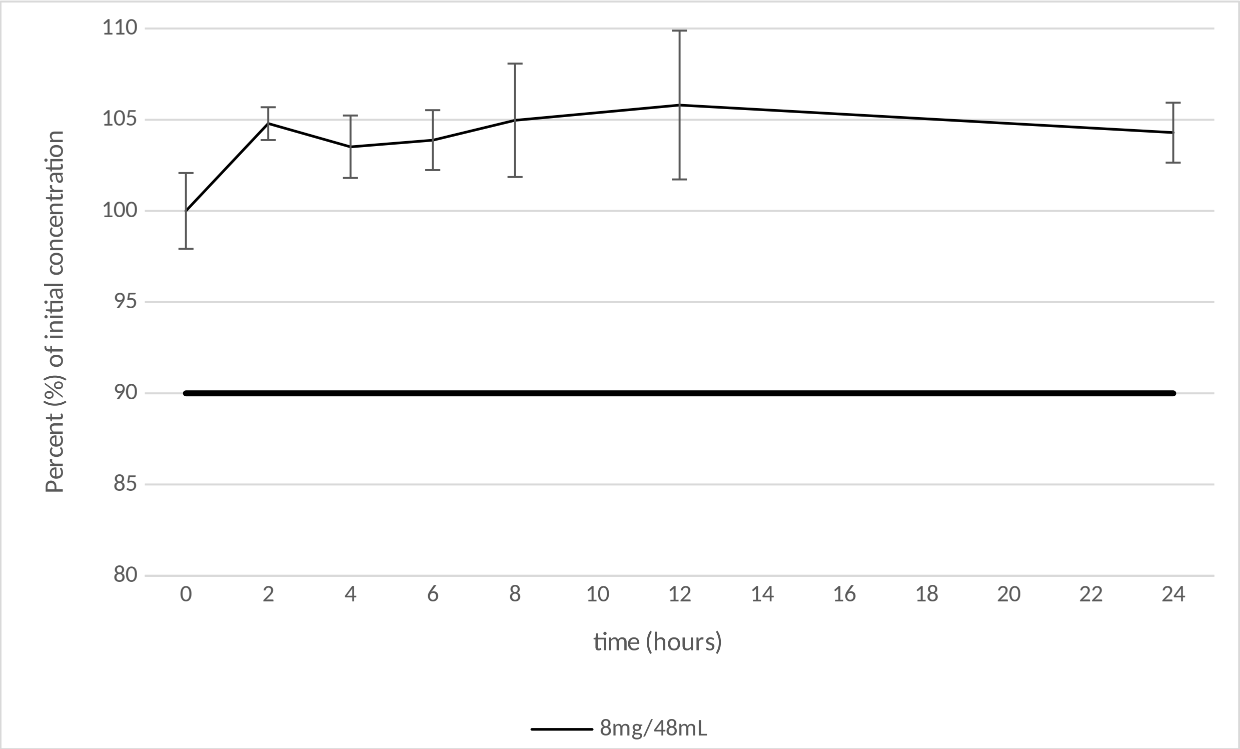
110
105
100
95
90
85
80
Percent (%) of initial concentration
0
2
4
6
8
10
12
14
16
18
20
22
24
time (hours)
8mg/48mL
